# Theoretical Investigation of Mono- and Di-Chloro-Substitient Effects on the Insulation and Greenhouse Properties of Octafluorocyclobutane

**DOI:** 10.3389/fchem.2016.00047

**Published:** 2016-12-15

**Authors:** Lin Cheng, Zhaoyu Qin, Chaohai Zhang, Huixuan Shi, Kun Zhao, Xiaoyu Xie, Haibo Ma

**Affiliations:** ^1^College of Electrical and Electronic Engineering, Huazhong University of Science and TechnologyWuhan, China; ^2^State Grid Electric Power Research InstituteWuhan, China; ^3^School of Electrical Engineering, Wuhan UniversityWuhan, China; ^4^School of Chemistry and Chemical Engineering, Nanjing UniversityNanjing, China

**Keywords:** insulating gas, density functional theory, octafluorocyclobutane, electron affinity, vibrational frequency

## Abstract

Octafluorocyclobutane, *c*-C_4_F_8_, and its derivatives are regarded as promising replacements of insulation gaseous SF_6_, which are currently widely used in electric equipment but suffer greatly from its greenhouse effect. Based on the recent finding that the dielectric and thermodynamics properties of insulating gases are greatly dependent on the molecule's microscopic electronic and vibrational parameters, in this work, we use density functional theory (DFT) to study the molecular structures, electron affinities, and IR-active vibrational frequencies as well as thermodynamic properties for *c*-C_4_F_8_ and a series of mono-, di-substituted *c*-C_4_F_8_ compounds. It is shown that DFT calculation of perfluoro-compounds is sensitive to the chosen functional. Although all chloro-substituted *c*-C_4_F_8_ molecules are found to have much larger electron affinities, only part of them have less IR intensity in the atmospheric IR “window” than *c*-C_4_F_8_. Such a study provides useful guideline for the pre-screening search for new insulation gases via electronic structure calculations.

## Introduction

Because of its excellent electrical insulationc performance and high stability, Sulfur hexafluoride (SF_6_) is nowadays the most widely used insulating gas in electric equipment [e.g., gas insulated switchgear (GIS)] in the world (Okubo and Beroual, 2011). However, the current usage of SF_6_ suffers a great limitation because it is a greenhouse gas (GHG) regulated under the Kyoto Protocol with a global warming potential (GWP) of 23,900 over 100-year time horizon (Fang et al., 2013). As a consequence, nowadays investigations for new replacement gases for SF_6_ becomes highly necessary.

Due to its strong dielectric and relative low global warming potential (GWP = 8700 = 36% GWP_SF6_) (Christophorou and Olthoff, 2001), Octafluorocyclobutane, c-C_4_F_8_, and its derivatives as well as mixtures are now regarded as promising insulation gaseous substitutes of SF_6_ (Itoh et al., 1991; Yamamoto et al., 2001; Yamaji and Nakamura, 2003; Yamaji et al., 2004; Wu et al., 2006; de Urquijo and Juárez, 2009; Li et al., 2014; Zhao et al., 2016). c-C_4_F_8_ has been known to have more superior dielectric properties than SF_6_. The sparkover voltage of c-C_4_F_8_ is about 1.3 times that of SF_6_ at atmospheric pressure for an ac waveform, and the lightning impulse voltage is 1.3–1.4 times that of SF_6_ (Yamamoto et al., 2001). The critical reduced electric field strength (*E*/*N*)_cr_, where *E* and *N* represent the electric field and the particle number density respectively, was determined to be 359~434 Td (Naidu et al., 1972; de Urquijo and Basurto, 2001; Liu et al., 2007), higher than that of SF_6_ (362 Td). At the same time, c-C_4_F_8_ suffers from a severe shortcoming that the liquefaction temperature of c-C_4_F_8_ (−8°C at 0.1 MPa) is much higher than that of SF_6_(−64°C at 0.1 MPa), which greatly limits its application in cold regions and high gas pressure GIS devices (Li et al., 2014).

Nowadays it is also well-known that the dielectric and thermodynamics properties of insulating gases are greatly dependent on the molecule's microscopic electronic and vibrational parameters. (Rabie et al., 2013; Zhang et al., 2016) The recent multiple regression analysis by us Zhang et al. (2016) and Rabie et al. (2013) have indicated that the gas's relative dielectric strength is proportional to the polarizability and electron affinity of the molecule and the liquefaction temperature is proportional to its polarizability and dipole moment. Therefore, for designing ideal insulation gas with high dielectric strength and low liquefaction temperature which can be widely used in electric equipment, molecules with small dipole moments, large electron affinities and balanced polarizability are expected to be selected through effective screening via electronic structure studies among a large number of candidate molecules. Of course the gas's greenhouse effect should be also examined by infrared spectroscopy investigations. In the past several years, there have been intensive studies on the structural, vibrational characteristics and the electron binding energies of c-C_4_F_8_ and its anion through Raman, infrared, photoelectron spectrums or electron-spin resonance (ESR) experiments as well as quantum chemical calculations. (Lemaire and Livingston, 1952; Bauman and Bulkin, 1966; Chang et al., 1971; Miller and Capwbll, 1971; Beagley et al., 1987; Mao et al., 1988; Purchase et al., 1997; Gallup, 2004; ElSohly et al., 2005; Bopp et al., 2007; Choi et al., 2013) However, very few research works on the electronic structures for c-C_4_F_8_ derivatives or their corresponding anions has been done, hindering the further rational design of new SF_6_ replacement gases.

Motivated by the above facts, in this work we presented a systematic study for the geometries, electron affinities, and vibrational frequencies as well as thermodynamic properties for the mono-, di-chloro-substituted *c*-C_4_F_8_ based on first principles density functional theory DFT (Hohenberg and Kohn, 1964; Kohn and Sham, 1965).

The rest of the paper is organized as follows. Computational details are reported in Section Computational Methods. In Section Results and Discussion, the ground state geometries, bonding energies, vibrational spectra, electron affinities, dipole moments, as well as thermodynamic properties are presented and analyzed. In Section Conclusions, the main points of this work are summarized and perspectives for future research are outlined.

## Computational methods

**Figure 1:**
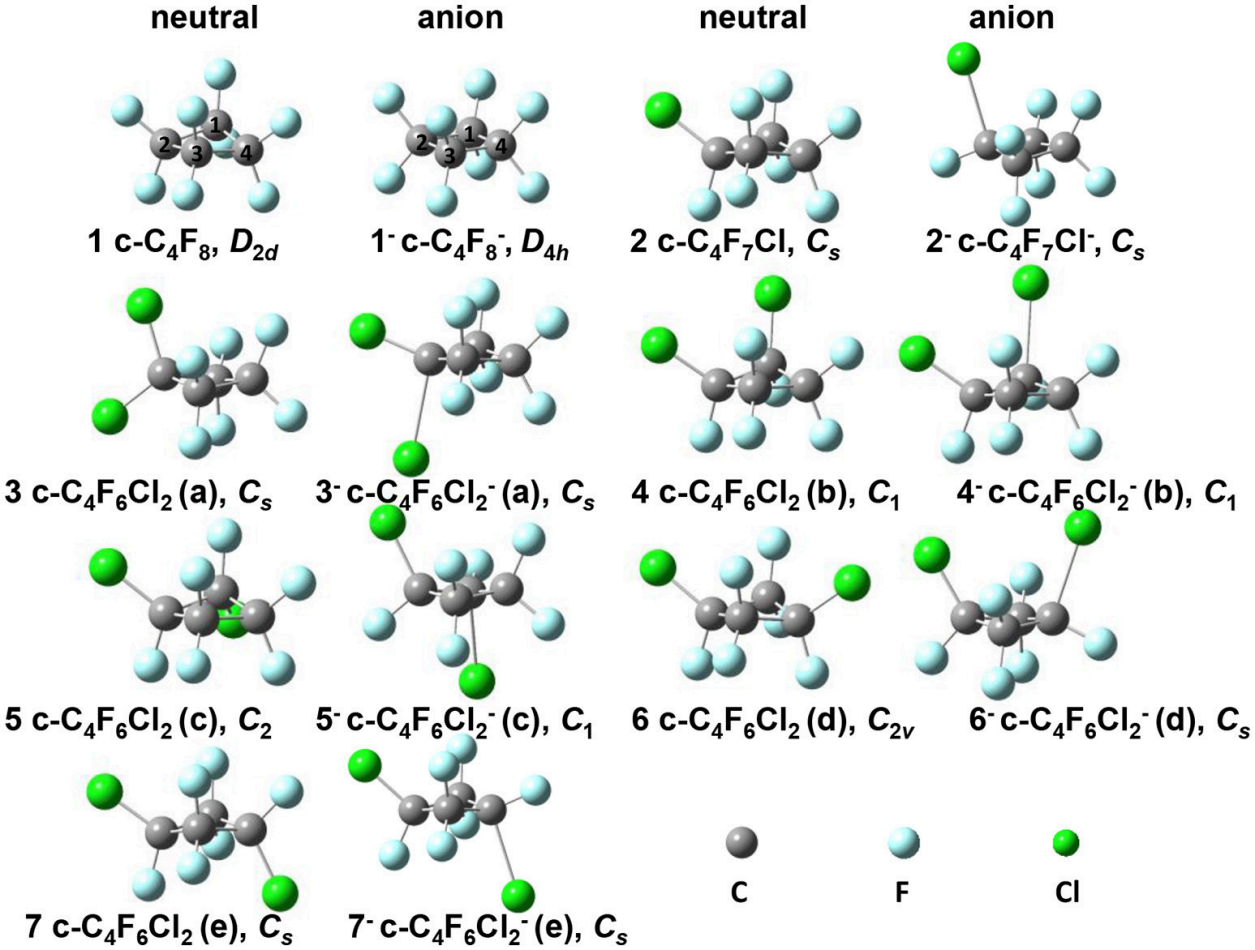
**Optimized geometries for neutral c-C_4_F_8−*n*_Cl_*n*_ (*n* = 0, 1, 2) series and their anions in their ground states**. 1: c-C_4_F_8_, *D*_2*d*_ symmetry; **1**^−^: c-C_4_${\text{F}}_{8}^{-}$, *D*_4*h*_ symmetry; **2**: c-C_4_F_7_Cl, *C*_*s*_ symmetry; **2**^−^: c-C_4_F_7_Cl^−^, *C*_*s*_ symmetry; **3**: c-C_4_F_6_Cl_2_(a), *C*_*s*_ symmetry; **3**^−^: c-C_4_F_6_${\text{Cl}}_{2}^{-}$(a), *C*_*s*_ symmetry; **4**: c-C_4_F_6_Cl_2_(b), *C*_1_ symmetry; **4**^−^: c-C_4_F_6_${\text{Cl}}_{2}^{-}$(b), *C*_1_ symmetry; **5**: c-C_4_F_6_Cl_2_(c), *C*_2_ symmetry; **5**^−^: c-C_4_F_6_${\text{Cl}}_{2}^{-}$(c), *C*_1_ symmetry; **6**: c-C_4_F_6_Cl_2_(d), *C*_2*v*_ symmetry; **6**^−^: c-C_4_F_6_${\text{Cl}}_{2}^{-}$(d), *C*_*s*_ symmetry; **7**: c-C_4_F_6_Cl_2_(e), *C*_*s*_ symmetry; **7**^−^: c-C_4_F_6_${\text{Cl}}_{2}^{-}$(e), *C*_*s*_ symmetry.

In this work, *c*-C_4_F_8_ (**1**) and its mono- and di-chloro-substituted derivatives (one mono-substitute c-C_4_F_7_Cl and five di-substitutes *c*-C_4_F_6_Cl_2_, see Figure 1) molecules (**2**-**7**) are selected and these molecules are studied using the first-principles DFT. DFT calculations are applied to optimize the molecule's geometry and describe its electronic structure of the selected molecules and corresponding negative ions, and frequency analysis is also performed. In our study, all the first-principle DFT calculations are performed using Gaussian09 D.01 software package (Frisch et al., 2009).

The electron affinities are evaluated as the difference of the molecule's energy in the following manner: the adiabatic electron affinity is determined by, EA_ad_ = *E*(optimized neutral) − *E*(optimized anion), the vertical electron affinity by, EA_vert_ = *E*(optimized neutral) − *E*(anion at optimized neutral geometry), and the vertical detachment energy of the anion by, VDE = *E*(neutral at optimized anion geometry) − *E*(optimized anion).

To benchmark the computational accuracy of our calculations for molecules with strong electronegative atoms like fluorine, seven DFT exchange-correlation functionals and four basis sets are tested, including GGA (Generalized gradient approximations) functional PBE (Perdew et al., 1996), hybrid functional B3LYP (Becke, 1993; Stephens et al., 1994) and M06 (Zhao and Truhlar, 2008), long-range corrected functional CAM-B3LYP (Yanai et al., 2004), ωB97-XD (Chai and Head-Gordon, 2008), and LC-ωPBE (Vydrov and Scuseria, 2006; Vydrov et al., 2006, 2007), as well as the newly developed meta-hybrid M06-2X (Zhao and Truhlar, 2008). The four tested basis sets include Pople basis sets (Krishnan et al., 1980; McLean and Chandler, 1980) 6–311+g(d) and 6–311+g(3df), and correlation-consistent basis sets aug-cc-pVTZ (Kendall et al., 1992) and aug-cc-pVQZ (Woon and Dunning, 1993).

## Results and discussion

### Benchmark tests for DFT functional and basis set

**Figure 2:**
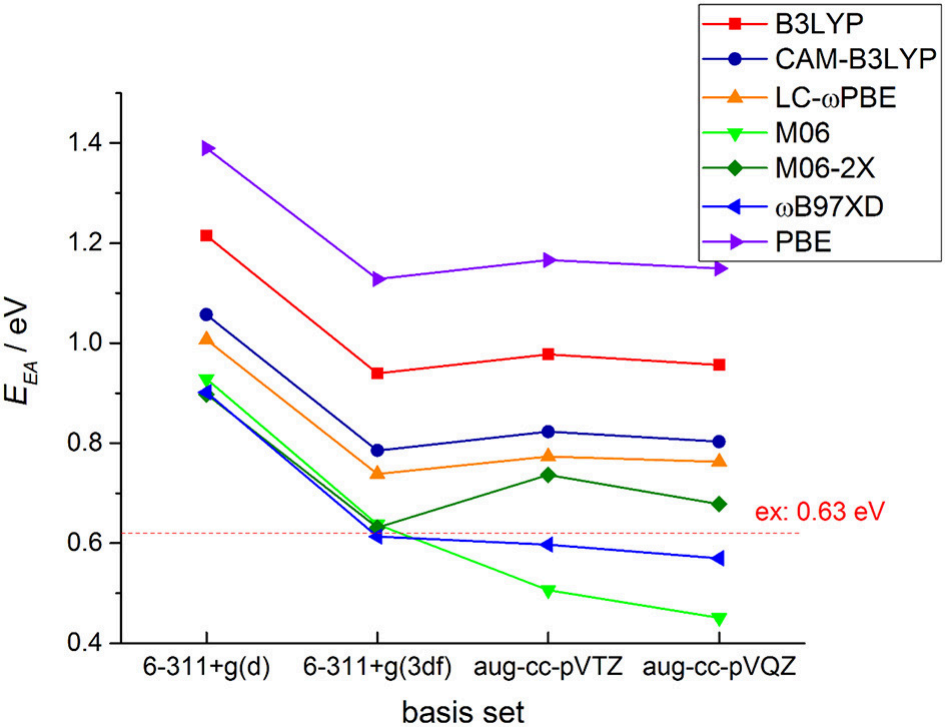
**Exchange-correlation functional and basis set test for DFT calculations of the adiabatic electron affinity energy (EA_ad_) for *c*-C_4_F_8_**.

It is well-known that electronic structure properties of the molecules, especially the electron affinity energy, are very sensitive to the DFT functionals and basis sets. In Figure 2, we plotted DFT calculated EA results with different functional and basis sets for *c*-C_4_F_8_ together with comparisons to experimental reference (0.63 ± 0.05 eV) (Chang et al., 1971). It is clearly shown that with basis set including *f* -type functions, long-range corrected or meta-hybrid functionals can generally give results much closer to experimental references than local or standard hybrid functions. Among them, M06-2X and ωB97-XD are shown to give smallest deviations (about −25 to 25% error). It is because in perfluoro-compounds, the large number of F atoms is associated with intense non-bonding interactions. Therefore, functional with long-range corrected or meta-hybrid functionals are required for the accurate calculation of the electronic structures of perfluoro-compounds.

As for the basis set, from Figure 2 one may also clearly notice that not only the diffused function but also *f* -type polarized function are necessary for an accurate theoretical prediction of EA for possible insulating gas molecules. It is shown that 6–311+g(d) basis set gives results with large deviations from experimental references for all of DFT functionals we adopted, while 6–311+g(3df), aug-cc-pVTZ and aug-cc-pVQZ basis sets can give more reasonable results. From the data shown in the Supporting Information, one can further notice that the basis set difference only causes slight distinctions in the optimized geometries and the EA difference by different basis sets mainly comes from the single point energy difference. Considering that the further incorporation of g-type (aug-cc-pVQZ) polarized function do not yield an obvious improvement of the calculated results, we finally decided to perform our further systematic DFT calculations with 6–311+g(3df) basis set to achieve a good compromise between accuracy and computational efficiency.

### Optimized geometries

The equilibrium structures of the optimized molecules (**1–7**) in their both neutral and anion forms obtained from geometry optimization by DFT calculation at M06-2X/6–311g+(3df) level were shown in Figure 1, and the key structural parameters are listed in Table 1. It is shown that the molecule symmetry is decreased from *D*_2*d*_ to *C*_2*v*_ or *C*_*s*_, *C*_2_ or even *C*_1_ with the increasing *n* for neutral forms of *c*-C_4_F_8−*n*_Cl_*n*_ (*n* = 0, 1, 2), due to the symmetry break by chlorine substitution. Interestingly we notice that while the geometry of neutral *c*-C_4_F_8_ (**1**) is bent with *D*_2*d*_ symmetry (∠C_2_C_1_C_3_C_4_ around 164.4°), that of its anion form (**1**^−^) becomes planar with *D*_4*h*_ symmetry (∠C_2_C_1_C_3_C_4_ being 180.0°). This finding is consistent with earlier ESR experiments (ElSohly et al., 2005) and theoretical calculations (Choi et al., 2013). The previous computational results have shown that the added electron to *c*-C_4_F_8_ is delocalized in a “pi-like” orbital extending over the entire molecule, strengthening the four C-C bonds via π-bonding interactions, and weakening the eight C-F bonds via σ*-antibonding interactions. However, such a bent-to-planar transition was not observed for *c*-C_4_F_8_ derivatives upon attachment of an electron, due to the decrease of the symmetry by mono- and di-chloro-substitutions.

**Table 1:** **Structural information of optimized molecules by DFT calculation at M06-2X/6–311g+(3df) level**.

**Molecule**	**Dihedral angle (∠0C_2_C_1_C_3_C_4_)/degree**			**r_*C*−*C*l_/Å**		
	**Neutral**	**Anion**	**Change percentage**	**Neutral**	**Anion**	**Change percentage**
**1**	164.4	180.0	9.5%	–	–	–
**2**	159.5	163.7	2.6%	1.73	2.41	39.3%
**3**	158.7	163.6	3.1%	1.74/1.75	1.71/2.48	−1.7%/41.7%
**4**	157.4	161.1	2.4%	1.73/1.74	1.78/2.38	2.9%/36.8%
**5**	157.3	161.7	2.8%	1.73/1.73	1.84/2.37	6.4%/37.0%
**6**	156.3	163.1	4.4%	1.73/1.73	1.76/2.40	1.7%/38.7%
**7**	158.0	162.0	2.5%	1.73/1.74	1.77/2.39	2.3%/37.4%

**Figure 3:**
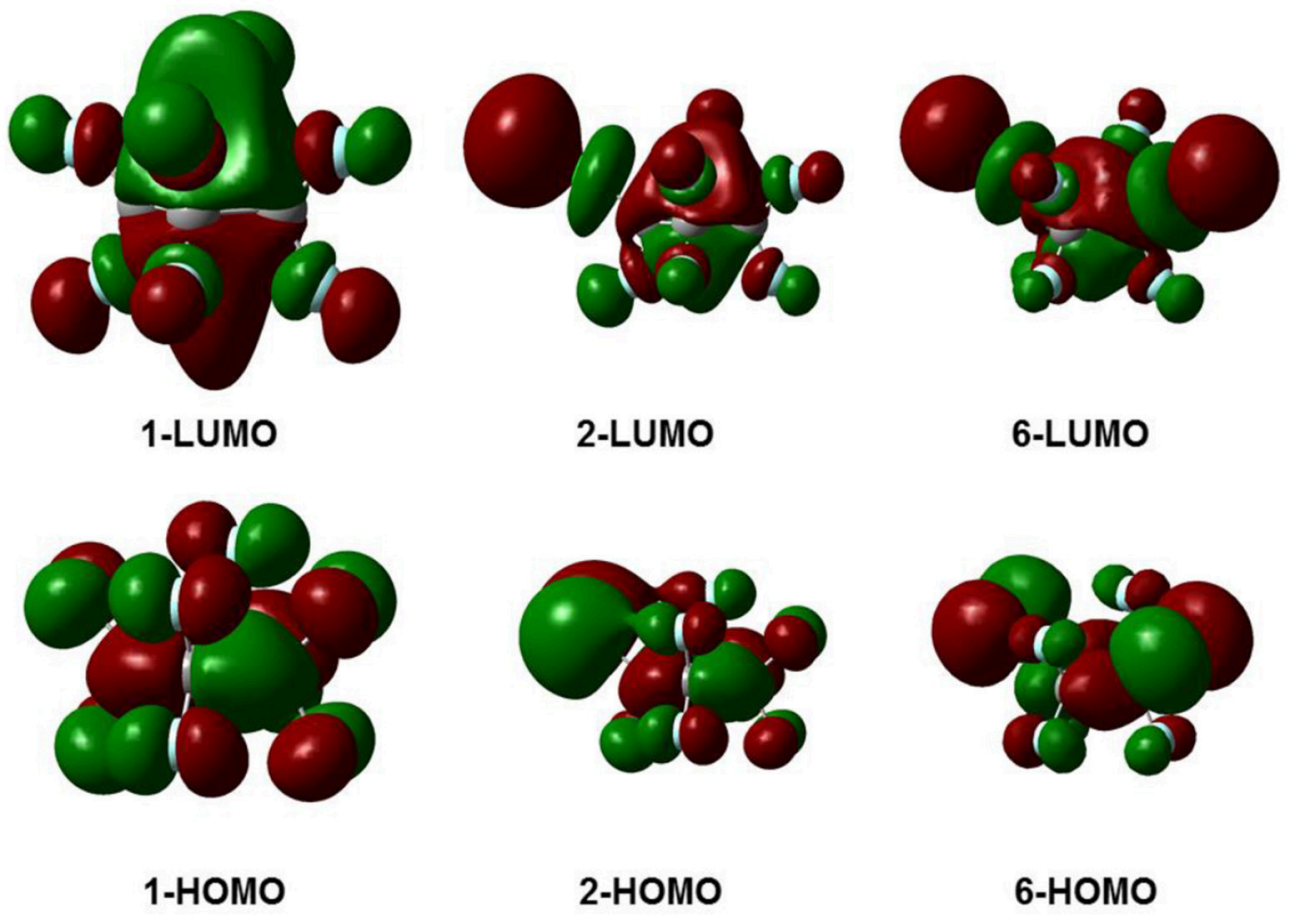
**The frontier molecular orbitals for selected typical molecules (1, 2, 6)**.

Figure 3 gives the highest occupied molecular orbitals (HOMO) and the lowest unoccupied molecular orbitals (LUMO) of the selected molecules 1, 2, and 6. For *c*-C_4_F_8_ (**1**), the LUMO is similar to a delocalized π-orbitals of the four carbon atoms, therefore, the attachment of an additional electron would make the carbon cycle more planar. On the contrary, for mono- and di-substituted molecules, the *p*-orbitals of chlorine atoms have much greater contributions to the LUMO, indicating a more localized feature at the Cl atom. As a consequence, the electron attached to the chloro-substitutes of *c*-C_4_F_8_ (**2**–**7**) will locate at the Cl atom to a great extent with an elongated C-Cl bond length and will not remarkably alter the planarity. This can be verified from the structure in Figure 1 and the data in Table 1, in which the ∠C_2_C_1_C_3_C_4_ change is less than 7° and the bond length difference between two C-Cl bonds are as large as 0.5–0.8 Å for the chloro-substitutes of *c*-C_4_F_8_ (**2–7**) upon electron attachment.

The results for binding energies and dipole moments for *c*-C_4_F_8−*n*_Cl_*n*_ (*n* = 0, 1, 2) are listed in Table 2. The *c*-C_4_F_8_ (**1**) molecule is found to have the highest binding energy as compared to its mono- and di-chloro-substituted derivatives (**2–7**), and a zero dipole moment due to its relatively high symmetry of *D*_2*d*_. At the same time, we notice that the binding energy is not sensitive to the different conformations for the isomers of *c*-C_4_F_6_Cl_2_ (**3–7**), but the dipole moment is found to be highly dependent on them. Because recent multiple regression analysis (Rabie et al., 2013; Zhang et al., 2016) have shown that the magnitudes of dipole moment are correlated to the liquefaction temperature of the gases, this implies that searching for new insulating gases which can work in extremely areas should carefully examine the molecule's possible different dipole moments caused by various isomerized conformations.

**Table 2:** **Binding energies and dipole moments of optimized molecules by DFT calculations at M06-2X/6–311g+(3df) level**.

**Molecule**	**Symmetry**	**Binding energy^a^/eV**	**Dipole moment/Debye**
**1**	*D*_2*d*_	54.09	0.000
**2**	*C_s_*	52.59	0.576
**3**	*C_s_*	51.23	0.650
**4**	*C*_1_	51.06	1.055
**5**	*C*_2_	51.08	0.575
**6**	*C*_2*v*_	51.08	0.751
**7**	*C_s_*	51.09	0.245

a *c-C_4_F_8−n_Cl_n_ → 4 C + (8-n) F + n Cl*.

### Electronic affinities

As the electron affinity feature has been revealed to be highly correlated with the insulating properties of the potential new insulating gases (Rabie et al., 2013; Zhang et al., 2016), in this work we also performed calculations for various electron affinity parameters of *c*-C_4_F_8−*n*_Cl_*n*_ (*n* = 0, 1, 2). The calculated EA_ad_, EA_vert_, and VDE by both ωB97-XD and M06-2X functionals are here listed in Table 3. As we have shown in Section Benchmark Tests for DFT Functional and Basis Set, by using suitable basis set (e.g., 6–311g+(3df)), DFT exchange-correlation functionals of ωB97-XD and M06-2X can yield very similar numerical results which are close to available experimental value. It is also shown that the electron affinity and VDA values are remarkably increased when the *c*-C_4_F_8_ (**1**) molecule is chloro-substituted. Considering that a larger EA value is beneficial to the improvement of relative electric strength (Rabie et al., 2013; Zhang et al., 2016), it would be useful to investigate the various halogen substitutions of currently used insulating gases of perfluoro-compounds for future improvement or replacement.

**Table 3:** **Adiabatic and vertical electron affinities of the neutral c-C_4_F_8_ and mono-, di-chloro-substituted c-C_4_F_8_ compounds as well as vertical detachment energies of their anions in units of eV**.

**Compound**	**Method**	**EA_ad_**	**EA_vert_**	**VDA**
**1**	ωB97-XD	0.61	−1.56	1.80
	M06-2X	0.63	−0.94	1.76
	Exp.	0.63^*a*^	–	–
**2**	ωB97-XD	1.30	−0.69	3.68
	M06-2X	1.27	−0.71	3.54
**3**	ωB97-XD	1.56	−0.35	3.85
	M06-2X	1.50	−0.50	3.71
**4**	ωB97-XD	1.32	−0.41	3.62
	M06-2X	1.27	−0.45	3.47
**5**	ωB97-XD	1.38	−0.49	3.61
	M06-2X	1.34	−0.51	3.45
**6**	ωB97-XD	1.31	−0.44	3.60
	M06-2X	1.28	−0.48	3.46
**7**	ωB97-XD	1.28	−0.48	3.62
	M06-2X	1.23	−0.51	3.47

a *From Chang et al. (1971)*.

### Vibrational frequencies and IR intensities

For the purpose of studying the greenhouse effect of the *c*-C_4_F_8_ and its derivatives, here we list the total infrared (IR) intensities and the cumulative IR intensities in the atmospheric IR “window” (800–1200 cm^−1^) in Table 4, the latter of which is widely used as a descriptor for qualitatively measuring the gas's greenhouse effect (Sturges et al., 2000). All these values can be calculated by frequency analysis using Gaussian09 D.01 package. It is clear that both the total IR intensity and cumulative IR intensity in the atmospheric IR “window” are increased upon the attachment of an electron for *c*-C_4_F_8−*n*_Cl_*n*_ (*n* = 0, 1, 2). Meanwhile, most chloro-substituted *c*-C_4_F_8_ compounds (**4**, **5**, **7**) show higher IR intensity in the atmospheric IR “window” than c-C_4_F_8_, implying a worse greenhouse effect. However, there are also some chloro-substituted c-C_4_F_8_ compounds (**2**, **3**, **6**) showing lower IR intensity in the atmospheric IR “window” than the *c*-C_4_F_8_ (**1**) molecule, implying a decreased greenhouse effect. Especially, a di-chloro-substituted *c*-C_4_F_8_ (**6**) has much less IR intensity (222.8 km × mol^−1^) in the atmospheric IR “window” than that of the *c*-C_4_F_8_ (**1**) molecule (411.4 km × mol^−1^). Therefore, new *c*-C_4_F_8_ derivatives like **6** can be expected to be possibly used as new insulating gases in electric equipment with much suppressed environmental problems.

**Table 4:** **IR intensities by DFT calculations at M06-2X/6–311g+(3df) level**.

	**Total IR intensity/km × mol^−1^**	**Cumulative IR intensity in the atmospheric IR “window”/km × mol^−1^**	**Percent/%**
**1**	1421.3	411.4	28.9
**1**^−^	3472.3	3347.4	96.4
**2**	1292.1	407.6	31.5
**2**^−^	2230.5	1077.3	48.3
**3**	1189.0	394.8	33.2
**3**^−^	2014.8	899.7	44.7
**4**	1167.6	451.4	38.7
**4**^−^	2089.5	855.9	41.0
**5**	1157.7	421.3	36.4
**5**^−^	2002.5	583.3	29.1
**6**	1132.8	222.8	19.7
**6**^−^	1888.9	848.1	44.9
**7**	1145.0	539.6	47.1
**7**^−^	1970.5	974.6	49.5

### Thermodynamic properties

Basic thermodynamic properties such as zero-point vibrational energy (ZPVE) and entropy as well as heat capacity have been also calculated for *c*-C_4_F_8−*n*_Cl_*n*_ (*n* = 0, 1, 2), and the results are presented in Table 5. It is shown that while all the translational, rotational and vibrational movements contribute considerably to the total entropy, only vibrational ones make the dominant contributions to the total heat capacity. It can be also noticed that the ZPVE decreases upon chloro-substitution, but the entropy and the heat capacity increase upon chloro-substitution.

**Table 5:** **Zero-point vibrational energy (ZPVE) and entropy as well as heat capacity of *c*-C_4_F_8_ and its mono-, di-chloro-substituted derivatives**.

**Molecules**		**1**	**2**	**3**	**4**	**5**	**6**	**7**
ZPVE/kcal × mol^−1^		30.8	29.6	28.5	28.4	28.4	28.4	28.4
Entropy/cal × mol^−1^×K^−1^	Total	95.0	101.0	102.2	102.0	101.3	101.5	102.3
	Trans.	41.8	42.0	42.2	42.2	42.2	42.2	42.2
	Rot.	27.4	30.6	31.0	31.0	29.7	29.7	31.0
	Vib.	25.8	28.3	28.9	28.8	29.4	29.6	29.1
Heat Capacity/cal × mol^−1^×K^−1^	Total	35.3	36.4	37.4	37.5	37.5	37.5	37.5
	Trans.	3.0	3.0	3.0	3.0	3.0	3.0	3.0
	Rot.	3.0	3.0	3.0	3.0	3.0	3.0	3.0
	Vib.	29.3	30.5	31.5	31.5	31.5	31.5	31.5

## Conclusions

In this work, we use DFT to investigate the molecular structures, electron affinities, and IR-active vibrational frequencies as well as thermodynamic properties for *c*-C_4_F_8_ and a series of mono-, di-substituted c-C_4_F_8_ compounds. It is shown that functional with long-range corrected or meta-hybrid functionals and f-component containing basis set are obligatory for DFT calculations of the electronic structures of perfluoro-compounds to reproduce the experimental results.

We found that the bending geometry (*D*_2*d*_ symmetry) of neutral *c*-C_4_F_8_ becomes planar (*D*_4*h*_ symmetry) upon the attachment of an electron as its LUMO delocalized in a “*p*-like” orbital extending over the entire molecule. On the contrary, the chloro-substituted *c*-C_4_F_8_ molecules remains non-planar upon electron attachment because their LUMOs have great contributions from the Cl atoms and become asymmetric.

Although all chloro-substituted c-C_4_F_8_ molecules are found to have much larger electron affinities, only part of them have less IR intensity in the atmospheric IR “window” than c-C_4_F_8_. This implies that new insulation gas with improved dielectric and environmental properties can be fabricated from some chloro-substituted *c*-C_4_F_8_ molecules after careful selection.

## Author contributions

KZ, XX, and HM designed the project. LC, ZQ, CZ, and HS did the calculations and analyzed the results. KZ, XX, and HM wrote the manuscript.

### Conflict of interest statement

The authors declare that the research was conducted in the absence of any commercial or financial relationships that could be construed as a potential conflict of interest.
