# Supplementary material for: Theoretical Investigation of Mono- and Di-Chloro-Substitient Effects on the Insulation and Greenhouse Properties of Octafluorocyclobutane
Source: Front Chem. 2016 Dec 15;4:47. doi: 10.3389/fchem.2016.00047 (PMC5156735; doi:10.3389/fchem.2016.00047)
Supplement: Supplementary file 1 [file DataSheet1.PDF]

Supporting Information

Theoretical Investigation of Mono- and Di-Chloro-Substitient Effects on the Insulation and Greenhouse Properties of Octafluorocyclobutane

Lin Cheng[1,2], Zhaoyu Qin[2,3], Chaohai Zhang[2], Huixuan Shi[1,2], Kun Zhao[2], Xiaoyu Xie[4,*], and Haibo Ma[4,*]

[1] *College of Electrical and Electronic Engineering, Huazhong University of Science and Technology, Wuhan 430074, China*

[2] *State Grid Electric Power Research Institute, Wuhan 430074, China*

[3] *School of Electrical Engineering, Wuhan University, Wuhan 430072, China*

[4] *School of Chemistry and Chemical Engineering, Nanjing University, Nanjing 210023, China*

I. MORE DETAILED INFORMATION OF THE BENCHMARK TESTS

A. basis sets dependence of the optimized conformations

TABLE I: Structural information of optimized molecules by DFT calculation at M06-2X level with different basis set.

Basis sets	Dihedral angle C_2-C_1-C_3-C_4 / degree		r_{C-C} / Å		r_{C-F} / Å	
	neutral	anion	neutral	anion	neutral	anion
6-311+g(d)	163.2	180.0	1.56	1.50	1.33	1.39
6-311+g(3df)	164.6	179.9	1.56	1.50	1.32	1.39
aug-cc-pVTZ	160.3	180.0	1.56	1.50	1.33	1.39
aug-cc-pVQZ	159.1	179.9	1.56	1.50	1.32	1.39

B. basis sets dependence of the electron affinity

TABLE II: EA calculation of optimized molecules (by DFT calculation at M06-2X/6-311+g(3df) level) using different basis set.

Basis sets	electron affinity / eV
6-311+g(d)	0.895
6-311+g(3df)	0.632
aug-cc-pVTZ	0.741
aug-cc-pVQZ	0.680

II. THE COORDINATES OF ALL MOLECULES AND ANIONS

The optimized coordinates of molecules and anions at M06-2X/6-311+g(3df) level(unit: Å) are displayed.

A. Molecules

1 C_4F_8

C	0.00000000	1.10007000	0.07432600
C	-1.10007000	0.00000000	-0.07432600
C	0.00000000	-1.10007000	0.07432600
C	1.10007000	0.00000000	-0.07432600
F	0.00000000	-1.64566700	1.28636800
F	-2.03927700	0.00000000	0.85691300
F	2.03927700	0.00000000	0.85691300
F	0.00000000	1.64566700	1.28636800
F	0.00000000	2.03927700	-0.85691300
F	-1.64566700	0.00000000	-1.28636800
F	0.00000000	-2.03927700	-0.85691300
F	1.64566700	0.00000000	-1.28636800

2 C_4F_7Cl

C	-0.88571600	0.20249700	0.00000000
C	0.21192000	-0.01438800	1.09412200
C	1.30934500	0.16545500	0.00000000
C	0.21192000	-0.01438800	-1.09412200
F	1.81055400	1.39664000	0.00000000
F	0.21252300	0.87351200	2.07305400
F	0.21252300	0.87351200	-2.07305400
F	-1.26278700	1.48991500	0.00000000
F	0.21192000	-1.24713800	1.58939400
F	2.28318300	-0.72992100	0.00000000
F	0.21192000	-1.24713800	-1.58939400
Cl	-2.24725400	-0.86585300	0.00000000

3 $C_4F_6Cl_2$ (a)

C	-0.78750400	0.06160400	0.00000000
C	0.32151600	-0.16259700	1.09048600
C	1.41529100	0.02809800	0.00000000
C	0.32151600	-0.16259700	-1.09048600
F	1.91461300	1.26047300	0.00000000
F	0.34131900	0.68703700	2.10222600
F	0.34131900	0.68703700	-2.10222600
F	0.32151600	-1.40903200	1.55429000
F	2.39466100	-0.86195400	0.00000000
F	0.32151600	-1.40903200	-1.55429000
Cl	-2.09573700	-1.08540700	0.00000000
Cl	-1.33599200	1.72200700	0.00000000

4 $C_4F_6Cl_2$ (b)

C	0.77941400	-0.39470700	0.41338800
C	-0.17378200	0.85776500	0.47038600
C	-1.33010300	-0.06084800	-0.04949500
C	-0.27280400	-1.11566400	-0.48459500
F	-2.07308800	-0.50416700	0.96098600
F	-0.35595800	1.32697100	1.70302100
F	-0.52809800	-2.37095700	-0.15952800
F	-2.10868000	0.42024500	-1.00407100
F	0.00697400	-1.02427200	-1.78118000
F	0.78191500	-0.97301000	1.62551600
Cl	2.39090700	-0.22847700	-0.19520100
Cl	0.22533200	2.13479600	-0.64014000

5 $C_4F_6Cl_2$ (c)

C	0.00631500	0.78447500	-0.49445600
C	-0.00631500	-0.78447500	-0.49445600
C	-0.22304400	-0.74676700	1.05296900
C	0.22304400	0.74676700	1.05296900
F	-1.49815600	-0.87820700	1.39939300
F	-0.52864900	1.58360200	1.74840300
F	0.52864900	-1.58360200	1.74840300
F	1.49815600	0.87820700	1.39939300
F	-1.21576200	1.26614000	-0.75817500
F	1.21576200	-1.26614000	-0.75817500
Cl	1.21576200	1.55753000	-1.46221600
Cl	-1.21576200	-1.55753000	-1.46221600

6 $C_4F_6Cl_2$ (d)

C	0.00000000	1.09713800	0.27013500
C	-1.08940000	0.00000000	0.04186000
C	0.00000000	-1.09713800	0.27013500
C	1.08940000	0.00000000	0.04186000
F	0.00000000	-1.46286700	1.56009100
F	2.08595700	0.00000000	0.90873500
F	1.56165000	0.00000000	-1.19851700
F	0.00000000	1.46286700	1.56009100
F	-1.56165000	0.00000000	-1.19851700
F	-2.08595700	0.00000000	0.90873500
Cl	0.00000000	2.47269500	-0.78263300
Cl	0.00000000	-2.47269500	-0.78263300

7 $C_4F_6Cl_2$ (e)

C	1.03926800	-0.06942000	0.00000000
C	-0.05379000	0.16732200	1.08849700
C	-1.16250300	-0.01932500	0.00000000
C	-0.05379000	0.16732200	-1.08849700
F	-0.04140900	-0.68146800	-2.10047000
F	-0.05379000	1.41551200	-1.54600300
F	1.39943200	-1.36215400	0.00000000
F	-0.05379000	1.41551200	1.54600300
F	-0.04140900	-0.68146800	2.10047000
F	-2.09801600	0.92989800	0.00000000
Cl	2.42018500	0.97495000	0.00000000
Cl	-1.86808400	-1.61012000	0.00000000

B. Anions

1^- $C_4F_8^-$

C	0.00000000	1.05914500	0.00043300
C	1.05914500	0.00000000	-0.00043300
C	0.00000000	-1.05914500	0.00043300
C	-1.05914500	0.00000000	-0.00043300
F	0.00000000	-1.93909600	-1.07393700
F	1.93861500	0.00000000	-1.07515300
F	-1.93861500	0.00000000	-1.07515300
F	0.00000000	1.93909600	-1.07393700
F	0.00000000	1.93861500	1.07515300
F	1.93909600	0.00000000	1.07393700
F	0.00000000	-1.93861500	1.07515300
F	-1.93909600	0.00000000	1.07393700

2^- $C_4F_7Cl^-$

C	-1.23487100	0.15751000	0.00000000
C	-0.14581300	0.35380000	1.08256400
C	0.85785400	0.24713600	0.00000000
C	-0.14581300	0.35380000	-1.08256400
F	2.02935700	0.87771500	0.00000000
F	-0.25265200	1.58019200	1.68748800
F	-0.25265200	1.58019200	-1.68748800
F	-2.19634400	1.09684100	0.00000000
F	-1.83637600	-1.03785000	0.00000000
F	-0.14581300	-0.52977800	2.09604000
F	-0.14581300	-0.52977800	-2.09604000
Cl	1.71850000	-2.00066400	0.00000000

3^- $C_4F_6Cl_2^-$ (a)

C	0.70371200	0.17469000	0.00000000
C	-0.31000200	0.29181800	1.08009000
C	-1.40461400	0.09371100	0.00000000
C	-0.31000200	0.29181800	-1.08009000
F	-1.99254600	-1.10486700	0.00000000
F	-0.31000200	-0.57882200	2.10025500
F	-0.31000200	-0.57882200	-2.10025500
F	-0.41226200	1.52390400	1.66455500
F	-2.37090000	1.02663800	0.00000000
F	-0.41226200	1.52390400	-1.66455500
Cl	2.22107300	0.96273600	0.00000000
Cl	1.31993900	-2.22271500	0.00000000

4^- $C_4F_6Cl_2^-$ (b)

C	0.01640400	0.91229600	0.40469200
C	-0.32409900	-0.48601800	0.73265600
C	0.85368400	-1.04215900	0.02736900
C	1.02009800	0.34768800	-0.63070400
F	1.93228300	-1.34452300	0.81885000
F	-0.72603600	-0.87152800	1.94017000
F	2.25281600	0.87179200	-0.54096900
F	0.72469300	-2.09440000	-0.79607300
F	0.64417300	0.41973700	-1.91037500
F	0.68287600	1.60015900	1.39686600
Cl	-1.21824900	2.04615100	-0.19544800
Cl	-2.25197200	-1.20038500	-0.47398200

5^- $C_4F_6Cl_2^-$ (c)

C	0.56327700	-0.70314500	-0.31465700
C	-0.49810100	-0.36712600	0.64020500
C	-0.27381300	1.09502000	0.52640900
C	0.59089400	0.78926600	-0.71909500
F	-1.31520600	1.92200300	0.34350900
F	0.00457500	1.05072900	-1.89421900
F	0.46618200	1.66799600	1.52451100
F	1.79164600	1.38645700	-0.73274400
F	0.33718200	-1.59208600	-1.30108200
F	-0.68890600	-0.99867000	1.79230400
Cl	2.15243500	-1.24861100	0.43559100
Cl	-2.60260000	-0.85797500	-0.34074900

6^- $C_4F_6Cl_2^-$ (d)

C	-1.10080200	0.18625600	-0.00009400
C	-0.17941700	-0.44178300	-1.07981200
C	0.78535300	-0.74238700	0.00024800
C	-0.17934800	-0.44087200	1.08016600
F	1.58347500	-1.80786800	0.00067800
F	-0.78219700	-1.52521100	1.66412300
F	0.18177900	0.34225200	2.10883200
F	-2.35143600	-0.32103500	0.00020400
F	0.18168900	0.34034500	-2.10923400
F	-0.78235200	-1.52671900	-1.66277800
Cl	-1.20474800	1.94357700	-0.00075400
Cl	2.48514000	0.94564900	-0.00039200

7^- $C_4F_6Cl_2^-$ (e)

C	1.02778600	0.10489100	0.00000000
C	-0.07758700	0.24751400	1.08159700
C	-1.06812600	0.06054700	0.00000000
C	-0.07758700	0.24751400	-1.08159700
F	-0.00496800	-0.63440500	-2.09332600
F	-0.07758700	1.47377100	-1.68892300
F	1.63622200	-1.09311200	0.00000000
F	-0.07758700	1.47377100	1.68892300
F	-0.00496800	-0.63440500	2.09332600
F	-2.28126400	0.60962300	0.00000000
Cl	2.27059500	1.36039700	0.00000000
Cl	-1.77268700	-2.22627900	0.00000000
